# Recent updates on anticancer mechanisms of polyphenols

**DOI:** 10.3389/fcell.2022.1005910

**Published:** 2022-09-29

**Authors:** Eshita Sharma, Dharam Chand Attri, Priyanka Sati, Praveen Dhyani, Agnieszka Szopa, Javad Sharifi-Rad, Christophe Hano, Daniela Calina, William C. Cho

**Affiliations:** ^1^ Department of Molecular Biology and Biochemistry, Guru Nanak Dev University, Amritsar, Punjab, India; ^2^ High Altitude Plant Physiology Research Centre (HAPPRC), HNB Garhwal University, Srinagar, Uttarakhand, India; ^3^ Graphic Era University, Dehradun, Uttarakhand, India; ^**4**^ Department of Biotechnology, Kumaun University, Nainital, Uttarakhand, India; ^5^ Chair and Department of Pharmaceutical Botany, Medical College, Jagiellonian University, Kraków, Poland; ^6^ Facultad de Medicina, Universidad del Azuay, Cuenca, Ecuador; ^7^ Department of Biological Chemistry, University of Orleans, Eure et Loir Campus, Chartres, France; ^8^ Department of Clinical Pharmacy, University of Medicine and Pharmacy of Craiova, Craiova, Romania; ^9^ Department of Clinical Oncology, Queen Elizabeth Hospital, Kowloon, Hong Kong SAR, China

**Keywords:** cancer, polyphenols, phytochemicals, migration, invasion, pharmacology, anticancer molecular mechanisms

## Abstract

In today’s scenario, when cancer cases are increasing rapidly, anticancer herbal compounds become imperative. Studies on the molecular mechanisms of action of polyphenols published in specialized databases such as Web of Science, Pubmed/Medline, Google Scholar, and Science Direct were used as sources of information for this review. Natural polyphenols provide established efficacy against chemically induced tumor growth with fewer side effects. They can sensitize cells to various therapies and increase the effectiveness of biotherapy. Further pharmacological translational research and clinical trials are needed to evaluate theirs *in vivo* efficacy, possible side effects and toxicity. Polyphenols can be used to design a potential treatment in conjunction with existing cancer drug regimens such as chemotherapy and radiotherapy.

## 1 Introduction

All happy healthy bodies are alike; every unhealthy human body is unhappy in its way. That is to say, the healthy cells of the human body collaborate on a structural and functional level to maintain the homeostasis and architecture of the body. Alteration of signaling pathways and mechanisms can influence the rate of cell proliferation and the risk of benign or malignant cell proliferation (Ali et al., 2022; Hossain et al., 2022; Javed et al., 2022). Transformed cells acquire new properties that can have a significant impact on local structures or the whole organism. Cells that proliferate acquire new properties (e.g., gain independence from the systems that control the progression of the cell cycle, and develop the ability to invade other tissues) that can alter the body’s homeostasis and predispose to pathological conditions that can have an unfortunate prognosis (Sharifi-Rad et al., 2021a; Jain et al., 2021; Kitic et al., 2022).

Cancer is one of the most prevalent diseases globally, affecting 10 million people (https://www.who.int/news-room/fact-sheets/detail/cancer). According to the WHO, cases will increase from 14 million to 27 million by 2050, with a global fatality rate of 17.5 million (Sharma et al., 2018; Briguglio et al., 2020). Cancer is a complicated phenotype with an infinite replicative capacity unaffected by growth cues (Zlatian et al., 2015; Buga et al., 2019). The capability of cancer cells to avoid cell death activation, persistent angiogenesis, tissue invasion, and metastasis is unrivalled (Docea et al., 2012; Mitrut et al., 2016). Considering this, metastasis is one of the primary causes of cancer-related mortality (Javed et al., 2022). However, a big part of cancer-related fatalities can be mitigated or averted by avoiding risk factors and following evidence-based preventative interventions (Hossain et al., 2022; Kitic et al., 2022). Several medications and therapies such as chemotherapy and radiation have been used to treat cancer (Rizvi et al., 2021b; Quetglas-Llabrés et al., 2022). However, these treatments and drugs have adverse effects, such as developing drug resistance in patients over time and non-specific toxicity to normal cells (Sharifi-Rad et al., 2021a; Dhyani et al., 2022). Nonetheless, these modern treatments can extend patients’ lives by weeks or months (GBD Colorectal Cancer Collaborators, 2022). As a result, developing a treatment that can achieve an objective measure of efficacy is critical; in this regard, plant-based drugs with established efficacy, fewer side effects, and safety are crucial (Sharifi-Rad et al., 2021c; Sharifi-Rad et al., 2021d; Sharifi-Rad al., 2022; Semwal et al., 2022). Plant polyphenols’ ability to protect against chemically induced and spontaneous tumor growth is well documented and deserves additional attention (Hadi et al., 2000; Hadi et al., 2010). The polyphenols, either in individuals or in groups, can be used to design treatment or combined therapy with distinct molecular mechanisms, ultimately resulting in greater efficacy (Azmi et al., 2006; Halliwell, 2007; Gupte and Mumper, 2009). Apart from their anti-cancer properties, polyphenols can also be used in conjunction with chemotherapy and radiotherapy (Asensi et al., 2011; Rizvi et al., 2021a).

**FIGURE 1:**
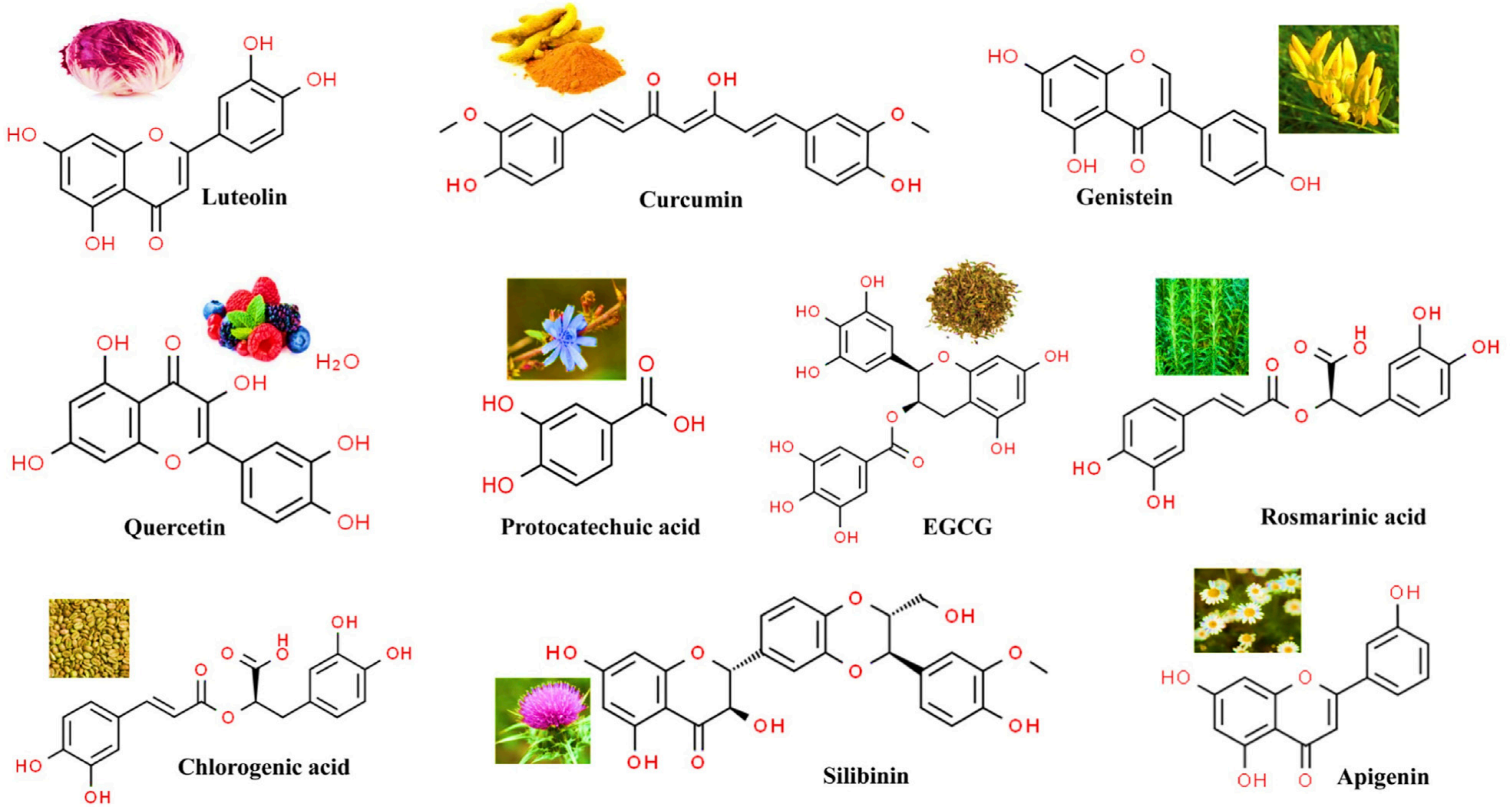
Chemical structures of most representative polyphenols employed as anticancer agents.

This updated review deals with different anti-cancer potential activities viz., Quercetin, Epigallocatechin, Curcumin, Silibinin, Apigenin, Luteolin, Genistein, Protocatechuic acid, chlorogenic acid and Eupatorine within several realms of existing knowledge on these molecules such as the mechanism of action and different promising scientific studies on their antitumor effect (Figure 1). This review will be a valuable resource for developing secondary metabolite-based anti-cancer therapy.

## 2 Methodology

In the current comprehensive review, the anticancer properties of the polyphenols presented according to their molecular mechanisms of action were analyzed. To compile this study, the papers published in the following specialized databases Web of Science, Pubmed/Medline, Google Scholar, Science Direct were studied. The following MeSH terms were used for the search: “Antineoplastic Agents/pharmacology”, “Antineoplastic Agents/therapeutic use”, “Apoptosis/drug effects”, “Cell Proliferation/drug effects”, “Chemoprevention”, “Cell Line, Tumor”, “Disease Models, Animal”, “Diet”, “Neoplasms/drug therapy”, “Neoplasms/prevention and control”, “Polyphenols/pharmacology”, “Polyphenols/therapeutic use”. *In vitro* and *in vivo* experimental pharmacological studies analyzed were processed to select the best data on the antitumor, anticancer, signaling and molecular anticancer mechanisms of polyphenols. The most important obtained data were summarized in tables and figures.

## 3 Cellular and molecular mechanisms of polyphenols in cancer: Current data from *in vitro* and *in vivo* studies

### 3.1 Anticancer mechanisms of quercetin

Quercetin exhibits multifactorial anti-tumor activity by decreasing cancer risk, growth, as well as the escalation of cancerous cells. Quercetin represses tumor growth of a variety of cancer cell lines, including breast, colorectal, head, lung, ovarian, melanoma, and leukemia, in a dose-dependent manner (Hashemzaei et al., 2017) (Table 1).

**TABLE 1:** Anticancer perspectives of polyphenols along with their mechanism of action and signaling pathway.

Compound	Cancer type	Model	Mechanism of action	Target	References
Quercetin	Thyroid	SW480	↑ apoptosis	Pro-NAG-1/GDF15	(Hong et al., 2021a; Hong et al., 2021b)
		HEK293			
		U2OS			
		BT-20 *in vitro*			
	Breast	MCF-7	↑ autophagy	Akt-mTOR	Jia et al. (2018)
		MDA-MB-231 *in vitro*			
		MCF-7 *in vitro*	↑ apoptosis	PI3K/Akt/mTOR	Li et al. (2018)
		MDA-MB-231 *in vitro*	↑apoptosis cell cycle arrest	Foxo3a	Nguyen et al. (2017)
		MDA-MB-231 *in vitro*	cell cycle arrest	Akt/AMPK/mTOR	(Rivera rivera et al., 2016; Lu et al., 2018)
	Oral	HCCLM3 *in vitro*	↓invasion	p-Akt1, MMP-2.MMP-9	Lu et al. (2018)
			↓migration		
		Male Syrian hamsters *in vivo*	↑apoptosis	NF-κB	Zhang et al. (2017)
	Liver	PC3	↑apoptosis cell cycle arrest	PI3K, AKT, ERK1/2	Erdogan et al. (2018)
		LNCaP *in vitro*			
	Ovarian	A2780S *in vivo*	↑ apoptosis	Caspase-3, -9, MCL-1, Bcl-2, Bax	Gao et al. (2012)
			↓ angiogenesis		
EGCG	Breast	MCF-7 *in vitro*	↓proliferation	P53 and Bcl-2	Huang et al. (2017)
			↓apoptosis		
		T47D *in vitro*	↓proliferation	PI3K/AKT	Moradzadeh et al. (2017)
			↑apoptosis		
		SUM-149	↑apoptosis	ER-α36	Pan et al. (2016)
		SUM-190			
		MDA-MB-231 *in vitro*			
	Ovarian	SKOV3 *in vitro*	↓proliferation	AQP5, NF-κB, p65, IκBα	Yan et al. (2012)
			↑apoptosis		
	Leukemia	NB4 *in vitro*	↓proliferation cell cycle regression	DNMT1, DNMT3a, DAPK1	Shi et al. (2018)
		NB4 *in vitro*	↑apoptosis	SHP-1-p38αMAPK-Bax	Gan et al. (2017)
		HL-60 *in vitro*	↑apoptosis	Bcl-2, caspase-3	Han and Kim, (2009)
	Lymphoma	BCBL-1	↑apoptosis ↑autophagy	MAPK	Tsai et al. (2017)
		BC-1 *in vitro*			
		Jeko-1	↑apoptosis	Fas, Bax, Bcl-2	Wang et al. (2015b)
		Raji *in vitro*			
	Renal	786-0ACHN *in vitro*	↓cell invasion	MMP-2, MMP-9	Chen et al. (2016)
		Caki-1 *in vitro*	↓suppression inactivation	Src, JNK	Sato et al. (2013)
	Head and neck	Tu686 *in vitro*	↑apoptosis	Bim, p21, p27, Bcl-2	Haque et al. (2015)
	Oral	C3H/HeJ	↑cell proliferation	HGF/c-Met	Koh et al. (2011)
		SCC VII/SF in vitro	↓migration		
			↓invasion		
		SCC-9 *in vitro*	↓cell growth	MMP-9	Chen et al. (2011)
			↓invasion		
	Thyroid carcinoma	TT TPC-1 ARO *in vitro*	↓angiogenesis	EGFR/RAS/RAF/MEK/ERK	Wu et al. (2019)
			↑apoptosis		
		8505C *in vitro*	↑suppression	TGF-β1/Smad	Li et al. (2019)
			↓ invasion		
			↓migration		
	Osteosarcoma	MG-63	↓cell proliferation cell cycle arrest	miR-1/c-MET	Zhu and Wang, (2016)
		U-2OS *in vitro*			
			↑apoptosis		
		MG63	↑apoptosis	miR-126	Jiang et al. (2014)
		U-2OS *in vitro*			
	Glioblastoma	GBM02	↑apoptosis ↑autophagy		Grube et al. (2018)
		GBM15			
		GBM16,17 *in vitro*			
	Hepatocellular carcinoma	HCC-LM3	↑cancer cell death	PFK	Li et al. (2016)
		Huh-7			
		HepG2			
		SMMC-7721 *in vivo*, *in vitro*			
Curcumin	Pancreatic	Patu8988	cell cycle arrest ↑apoptosis	YAP/TAZ	Zhou et al. (2016)
		Panc-1 *in vitro*			
	Glioblastoma	A172 *in vitro*	↑autophagy		Lee et al. (2019)
	Breast	MCF-7 *in vitro*	↑apoptosis	Mcl-1	Koohpar et al. (2015)
Silibinin	Colorectal	CT26 *in vitro*	↓angiogenesis ↑autophagy	Bax, Caspase-3, COX-2	Sameri et al. (2021)
			↑apoptosis ↓migration		


	Glioblastoma	A172 *in vitro*	↑apoptosis	mTOR, YAP	Bai et al. (2018)
			↓autophagy		
	Breast	MCF7 *in vitro*	↑cell autophagy	BNIP3	Jiang et al. (2015)
	Ovarian	SKOV-3 *in vitro*	↑ apoptosis	P53, P21	Pashaei-asl et al. (2018)
		A2780 SKOV3 *in vitro*, *in vivo*	↓tumor growth	p-ERK, p-Akt	Cho et al. (2013)
Apigenin	Cervical	HeLa *in vitro*	↓cell-renewal capability	CK2α	Liu et al. (2015)
	Breast	A549 *in vitro*	↓proliferation	PI3K/Akt	Zhou et al. (2017)
			↑migration ↓invasiveness		

		MDA-MD-231 *in vitro*, *in vivo*)	cell cycle arrest	cyclin A, cyclin B, CDK1, p21WAF1/CIP1	Tseng et al. (2017)
		MDA-MB-468, 4T1 *in vitro*	↑immune response	IFN-γ, PD-L1, STAT1	Coombs et al. (2016)
		SKBR3 *in vitro*	↑apotosis	p-JAK2 and p-STAT3, VEGF	Seo et al. (2015)
	Lung	H1299 H460 *in vitro*	↓cell proliferation	GLUT 1	Lee et al. (2016b)
			↑apoptosis		
	Prostate	PC3 *in vitro*	cell cycle arrest	p21 and p27; caspases-8,-3, TNF-α	(Shukla et al., 2015; Erdogan et al., 2016)
			↑apoptosis		
		DU145 *in vitro*	cell cycle arrest	IKK—IκBα	Shukla et al. (2015)
			↑apoptosis		
			↓tumorigenesis		
		PC3-M LNCaP C4-2B *in vitro*	↓cell proliferation ↓metastases	Smad2/3, Src/FAK/Akt	Mirzoeva et al. (2014)

	Colorectal	SW480 *in vitro*	↓cell proliferation	Wnt/β-catenin	Xu et al. (2016)
			↓migration		
			↓invasiveness		
		DLD1	↓cell proliferation ↓migration	NEDD9	Dai et al. (2016)
		SW480 *in vitro*, *in vivo*			
			↓invasiveness		
		HCT116 *in vitro*	↓proliferation	cyclin B1, Cdc2, Cdc25c	Lee et al. (2014)
			↑apoptosis		
			↑autophagy		
	Renal cell	ACHN	cell cycle arrest	p53	Meng et al. (2017)
		786–0			
		Caki-1 *in vitro*, *in vivo*			
	Head and neck squamous carcinoma	HSC-3	↓cancer cell markers	CD44, NANOG, CD105	Ketkaew et al. (2017)
		HN-8			
		HN- 30 *in vitro*			
Luteolin	Prostate	PC3	↑apoptosis	miR-301	Han et al. (2016)
		LNCaP *in vitro*			
	Hepatocellular carcinoma	SMMC-7721 *in vitro*	↑apoptosis	BL-2, caspase-8	Cao et al. (2017)
			↑autophagy		
	Cholangiocarcinoma	n CCA	cell cycle arrest	JAK/STAT3	Aneknan et al. (2014)
		KKU-M156 *in vitro*	↑apoptosis		
			↓migration		
	Pancreatic	BxPC-3 *in vitro*	↓cell proliferation	GSK-3β, NF-κB, p65	Johnson and Mejia, (2013)
	Colon	Balb/C mice *in vivo*	↑apoptosis	MMP-2, -9, TIMP-2	Pandurangan et al. (2014)
	Oral	CD44 *in vitro*	↓invasiveness clonogenicity	IL-6/STAT3	Tu et al. (2016)
	Gastric	MKN45	↑mirnas expression	Notch1, PI3K, AKT, mTOR, ERK, STAT3	Pu et al. (2018)
		BGC823 *in vitro*, *in vivo*			
		BGC-823	↑apoptosis	miR-34a, Bcl-2	Wu et al. (2015)
		SGC-7901 *in vitro*			
	Neuroblastoma	SH-SY5Y *in vitro*	cell cycle arrest		Wang et al. (2015a)
			↑apoptosis		
	Breast	MDA-MB-231 *in vitro*, *in vivo*	apoptosis	STAT3	Yang et al. (2014)
	Glioblastoma	U87MG	↑apoptosis	miR-7-1-3p	Chakrabarti and Ray, (2016)
		T98G *in vivo*	↑autophagy		
Genistein	Breast	MCF-7	cell cycle arrest		Jiang et al. (2018)
		MDA-MB-231 *in vitro*	↓proliferation		
			↑inhibition		
	Hepatocellular	Hepa1-6 *in vitro*	↑apoptosis		Sanaei et al. (2018)
			↓proliferation		
	Cervical	HeLa *in vitro*	↑apoptosis	GRP78, CHOP	Yang et al. (2016b)
	Liver	HepG2 *in vitro*	↑apoptosis cell cycle arrest	Bax, Bcl-2, caspase-3, 9	Zhang et al. (2019)
Protocatechuic acid	Gastric carcinoma	B16/F10 *in vitro*	↓cell migration	Ras/Akt/NF-kB	Lin et al. (2011)
			↓invasion		
	Breast	MCF-7	↑apoptosis	Caspase-3, 9	Yin et al. (2009)
		A549	↓metastasis		
		HepG2	↓invasion		
		HeLa			
		LNCaP *in vitro*			
Rosmarinic acid	Colorectal	CT26 *in vitro*	cell cycle arrest	EMT, MMPs, AMPK	Han et al. (2018a)
			↑apoptosis		
	Pancreatic	Panc-1	↓cell viability	miR-506, MMP2/16	Han et al. (2019)
		SW1990 *in*	↓growth		
		*vitro*	↓invasion		
			↓migration		
	Human melanoma	A375 *in vitro*	↑apoptosis	ADAM17/EGFR/AKT/GSK3β	Huang et al. (2021)
			↓proliferation		
			↓migration		
	Breast	Mice	↓inflammation	NF- κB, p53 caspase-3	Mahmoud et al. (2021)
		*in vivo*	↓angiogenesis		
			↑apoptosis		
	Head and Neck Squamous Carcinoma	UM- SCC-6	↓cell viability	MAPK/ERK	Tumur et al. (2015)
		UM-SCC-	↓ migration		
		10B *in vitro*	ROS		
	Colon	Wistar rats *in vivo*	↓cell proliferation	IL-6, COX-2, p65	Karthikkumar et al. (2015)
	Gastric	MKN45 *in vitro*	↓tumorgenesis	miR-155, IL-6/STAT3	Han et al. (2015)
Chlorogenic Acid	Hepatocellular carcinoma	Hep-G2	↑apoptosis	MAPK, NF-κB, TGF-β	Jiang et al. (2021)
		Huh-7 *in vitro*			
	Breast	Females *in vivo*	↑apoptosis ↓metastasis	NF-κB/EMT	Zeng et al. (2021)
		4T1 *in vitro*	↑apoptosis	Bax and Bcl-2	Changizi et al. (2020)
	Tumor cells	HepG2 A549 *in vitro*	↓migration	mTORC2/F-actin	Tan et al. (2019)
			↓invasiveness		
			↓tumor growth		
	Glioblastoma	G422 *in vitro*	↑apoptosis	LPS/IFNγ	Xue et al. (2017)
	Leukemia	U937	↑apoptosis		Liu et al. (2013)
		K562 *in vitro*	↓proliferation		
Eupatorin	Breast	4T1 *in vivo*	↓tumor development	MMP-9, NF-κB, NK1.1, CD8+	Abd Razak et al. (2020)
			↓metastasis		
		MCF-7	Cell cycle arrest, invasion, migration	Phospho-Akt	Razak et al. (2019)
		MDA-MB-231 *in vitro*			
		MDA-MB-468 *in vitro*	cell cycle arrest	CYP1	Androutsopoulos et al. (2008)
	Cervical	HeLa *in vitro*	cell cycle arrest	p53, p21, Bax	Lee et al. (2016a)
			↑apoptosis		
	Leukemia	HL-60	↑apoptosis	c-jun N-terminal kinases	Estevez et al. (2014)
		U937			
		Molt-3 *in vitro*			

Symbols: ↑increase, ↓decrease. Abbreviations: ADAM17/EGFR/AKT/GSK3β, a disintegrin and metalloproteinase-17/epidermal growth factor receptor/protein kinase B/Glycogen synthase kinase3β; Akt/AMPK/mTOR, protein kinase B/Adenosine monophosphate-activated protein kinase/mammalian target of rapamycin; Akt-mTOR, protein kinase B/mammalian target of rapamycin; AQP5, aquaporin-5; Bax, Bcl2-Associated X Protein; Bcl-2, B-cell leukemia/lymphoma 2 protein; Bim, B cell lymphoma-2-like 11; BL-2, Burkitt’s lymphoma-2; CD8^+^, cluster of differentiation 8; Cdc2, cell-division cycle 2; CD44, cluster of differentiation 44; CD105, endoglin; CDK1, cyclin-dependent kinase-1; CHOP, cyclophosphamide-hydroxydaunorubicin-oncovin-prednisone; CK2α, catalytic subunit of protein kinase CK2; COX-2, cyclooxygenase-2; CYP1, cytochrome P450 1; DAPK1, death associated protein kinase 1; DNMT1, DNA (cytosine-5)-methyltransferase 1; DNMT3a, DNA methyltransferase 3 alpha; EGFR/RAS/RAF/MEK/ERK, epidermal growth factor receptor/rat sarcoma virus/rapidly accelerated fibrosarcoma/mitogen-activated extracellular signal-regulated kinase/extracellular signal-regulated kinase; EMT, epithelial-mesenchymal transition; ERK1/2, extracellular signal-regulated kinase 1/2; ER-α36, estrogen receptor-alpha36; Fas, cell surface death receptor; Foxo3a, forkhead box class O 3a; GLUT 1, glucose transporter 1; GRP78, glucose-regulating protein 78; GSK-3β, glycogen synthase kinase-3 beta; HGF/c-Met, hepatocyte growth factor/c-mesenchymal-epithelial transition factor; IFN-γ, interferon-gamma; IκBα, nuclear factor of kappa light polypeptide gene enhancer in B-cells inhibitor, alpha; IKK–IκBα, IκB kinase-nuclear factor of kappa light polypeptide gene enhancer in B-cells inhibitor; IL-6, interleukin-6; JNK, c-Jun N-terminal kinase; LPS/IFNγ, lipopolysaccharide/interferon-gamma; MAPK, mitogen-activated protein kinase; MCL-1, myeloid cell leukemia 1; miR-1/c-MET, microRNA-1/mesenchymal-epithelial transition factor; miR-126, microRNA-126; miR-301, microRNA-301; MMPs, matrix metalloproteinases; mTORC2/F-actin, mTOR complex 1/filamentous actin; NEDD9, neural precursor cell expressed, developmentally downregulated nine; NF-κB, nuclear factor kappa B; NK1.1, natural killer 1.1; p-ERK, phosphorylated-extracellular signal-regulated kinase; PD-L1, programmed death-1; PFK, phosphofructokinase; pro-NAG-1/GDF15, pro-nonsteroidal anti-inflammatory drug-activated gene-1/growth differentiation factor 15; p21WAF1/CIP1, wild-type activating fragment-1/cyclin-dependent kinase inhibitory protein-1; PI3K/Akt/mTOR, phosphatidylinositol-3-kinase/protein kinase B/mammalian target of rapamycin; p-Akt1, phosphorylated-serine/threonine kinase 1; p-JAK2, phosphorylated Janus kinase 2; p-STAT3, phosphorylated signal transducer and activator of transcription 3; SHP-1-p38αMAPK-Bax, Src homology 2 domain-containing protein tyrosine phosphatase 1 mitogen-activated protein kinase; Src, Proto-oncogene tyrosine-protein kinase; STAT1, signal transducer and activator of transcription 1; TGF-β1/Smad, transforming growth factor-β1/suppressor of mothers against decapentaplegic; TIMP-2, tissue inhibitor of metalloproteinase-2; TNF-α, tumour necrosis factor-alpha; VEGF, vascular endothelial growth factor receptor; Wnt, wingless-related integration site; YAP/TAZ, yes-associated protein/transcriptional coactivator with PDZ-binding motif.

Many experimental pharmacological studies have revealed that quercetin exerts the anti-tumor effect by changing the cell cycle, metastasis progression, angiogenesis, cell proliferation inhibition, and promotion of apoptosis, thus, affecting autophagy (Tang et al., 2020). Additionally, the anti-tumor activity of quercetin involves the regulation of epigenetics in tumor cells, which further regulate mRNA expression, and DNA methylation. Therefore, it increases the bioavailability of the drug to the tumor cell by the inhibition of BCRP, MRP1, and P-gp (P-glycoprotein) (Kedhari Sundaram et al., 2019; Panthi et al., 2020).

Anti-tumor property of quercetin is well depicted by its ability to interfere in various cellular signal pathways and inhibits enzymes responsible for carcinogens activation (Rauf et al., 2018; Panthi et al., 2020). Quercetin causes arrest at the G2/M or G1 phase in different cell types. Moreover, quercetin-mediated apoptosis results from the induction of stress proteins, microtubule disruption, and cytochrome *c* release from mitochondria, followed by caspases activation (Panthi et al., 2020). Quercetin triggers arrest of the G2/M phase in human esophageal squamous cell carcinoma at mRNA plus protein levels via up and downregulation of p73, p21waf1, and cyclin B1, respectively. Quercetin reduces the propagation of cancer cells via the inhibition of intracellular signaling, i.e., PI3K, EGFR, and Her2/neu. Quercetin induces apoptosis of cancer cells by modulating the modulation of survival signaling pathways (Akt, NF-*k*B) or regulatory molecules, which are linked to cell death (Nguyen et al., 2017) (Table 1).

#### 3.1.1 *In vitro* studies

**FIGURE 2:**
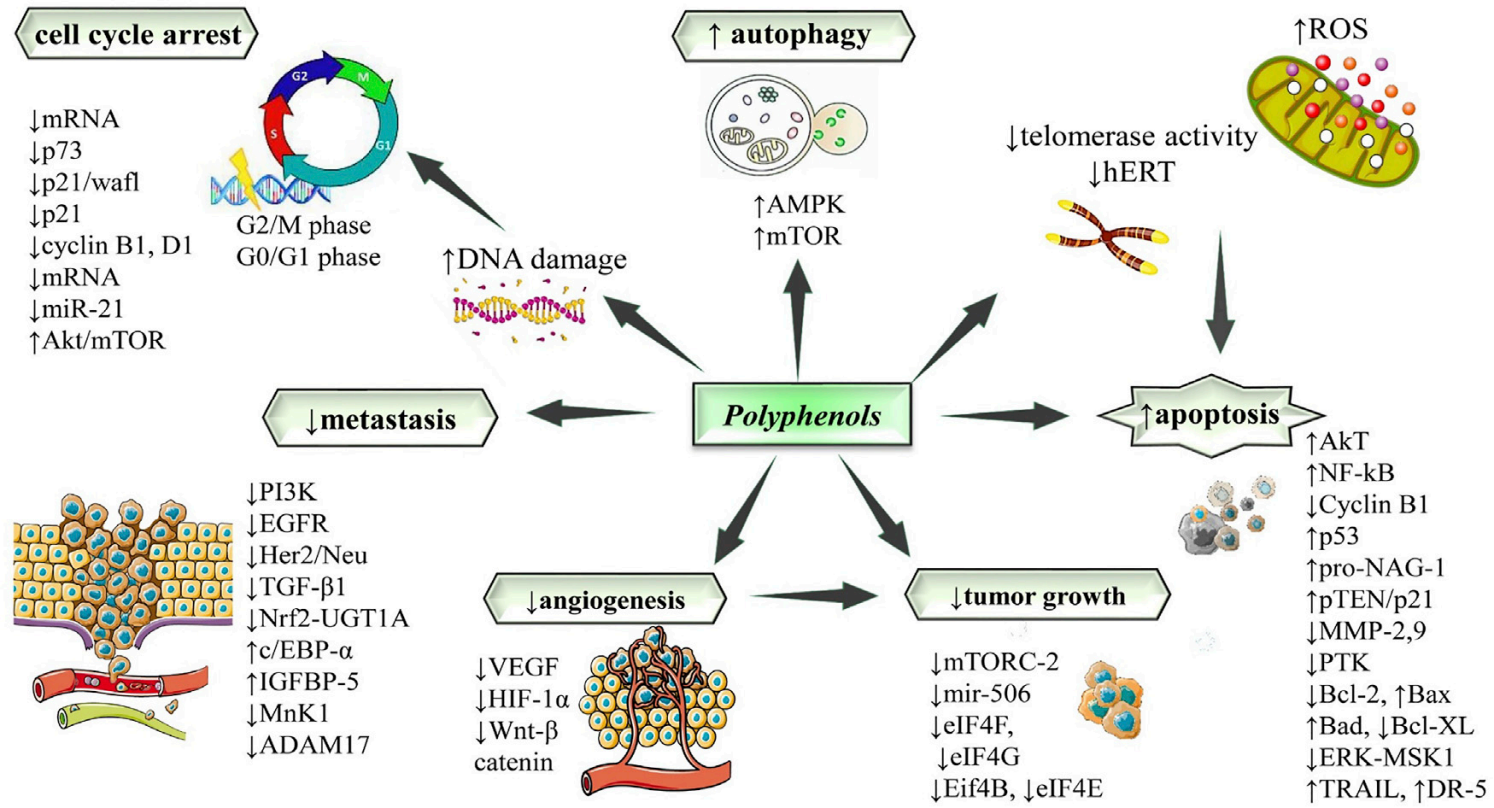
Potential molecular targets and signaling pathways for the antitumor effect of polyphenols. Symbols: ↑increase, ↓decrease. Abbreviations: ADAM17, ADAM metallopeptidase domain 17; Akt/mTOR, protein kinase B/mammalian target of rapamycin; AMPK, adenosine monophosphate-activated protein kinase; BAD, Bcl-2 antagonist of cell death; Bax, Bcl2-Associated X Protein; Bcl-2, B-cell leukemia/lymphoma 2 protein; Bcl-xL, B-cell lymphoma-extra large; c/ebp-α, CCAAT/enhancer-binding protein-alpha; DR-5, death receptor 5; EGFR, epidermal growth factor receptor; ERK/MSK, extracellular signal-regulated kinase/mitogen- and stress-activated kinase 1; Her2/Neu, human epidermal growth factor receptor 2/neutrophills; HIF-1-alpha, hypoxia-inducible factor 1-alpha; IGFBP-5, insulin-like growth factor-binding protein-5; hTERT, human telomerase reverse transcriptase; miR, microRNA; MMP, matrix metalloproteinase; MnK-1, a family of serine/threonine kinases; mRNA, messenger ribonucleic acid; mTOR, mammalian target of rapamycin; mTORC2, mTOR Complex 2; NF-κB, nuclear factor kappa B; Nrf1, nuclear respiratory factor 1; PI3K: phosphatidylinositol 3-kinase; pro-NAG-1, pro-nonsteroidal anti-inflammatory drug-activated gene-1; pTEN, phosphatase and TENsin homolog deleted on chromosome 10; PTK, protein tyrosine kinase; TGF-β1, transforming growth factor-β1; TRAIL, tumor-necrosis factor related apoptosis-inducing ligand; UGT1A, UDP glucuronosyltransferase 1 family; VEGF, vascular endothelial growth factor receptor; Wnt, wingless-related integration site.

Various *in-vitro* and xenograft models have shown the inhibitory role of quercetin in the growth of cancers which are revealed in apoptosis promotion, inhibition of proliferation, angiogenesis, and metastasis, making it a potential antitumor agent (Panthi et al., 2020; Tang et al., 2020) (Table 1). Quercetin possesses its antitumor activity in a dose varied from 3 to 50 µM on numerous cell lines, i.e., 3.5 μM for the B16-BL6 murine cancer cell line, PC-3 and DU-145 human prostate cancers were inhibited at 25 μM and in human breast carcinoma cells confirmed inhibition at 10 μM (Gibellini et al., 2011; Panthi et al., 2020). The ability to trigger apoptosis of quercetin in cancerous cells makes this molecule an appealing preference for various cancer therapies (Figure 2). Quercetin arrests the cell cycle and induces apoptosis in conjunction with p53 phosphorylation, which stabilizes p53 at mRNA and protein levels in HepG2 cells. In HCT116 colon carcinoma cells, apoptosis of the tumor cells is caused by p53 to the quercetin-mediated expression of NAG-1 (Gibellini et al., 2011). However, a recent study has revealed that quercetin induces apoptosis through only pro-NAG-1 expression; however, mature NAG-1 did not induce apoptosis which was mediated by the C/EBP transcription factor in thyroid cancer cell lines (Hong et al., 2021a) (Table 1).

H1299 lung carcinoma cell line was discovered to have extra liable to cytotoxicity compared to the A549 lung carcinoma line (Gibellini et al., 2011). Quercetin exerted significant inhibition of cell proliferation and induction of apoptosis in human renal cell adenocarcinoma cells via the survivin mRNA inhibition, protein expression, and caspase 3 activation (Reyes-Farias and Carrasco-Pozo, 2019). The proteasomal degradation of survivin takes place by quercetin, which in flip increases the concentration of cyclin B1 and p53 proteins, thereby enhancing surviving and p21 protein expression and inhibiting apoptosis (Gibellini et al., 2011).

In prostate cancer cells, quercetin complements the TNF-associated apoptosis-inducing ligand (TRAIL), which in turn causes apoptosis through induction of the expression of the death receptor (DR)-5. The upregulation of DR-5 and downregulation of β-FLIP make contributions to the recuperation of TRAIL sensitivity which further inhibits hepatocellular carcinoma cells by the use of quercetin. The superior TRAIL-caused apoptosis using quercetin takes vicinity via inhibiting the expression of the ERK-MSK1 pathway (Gibellini et al., 2011).

In a recent study, the expansion of ovarian cancer cells was inhibited by quercetin by a mechanism related to TGF-β1 (Shafabakhsh and Asemi, 2019). TGF-β1 possesses potent hematopoietic regulatory properties, depending on their stage progenitor differentiators; it either stimulates or inhibits the noncancerous myeloid progenitor’s growth. Quercetin inhibits the cell cycle and promotes apoptosis in breast cancer and leukemia cell xenograft models via Akt/mTOR pathway in a dose-dependent manner (Rivera rivera et al., 2016) (Table 1).

#### 3.1.2 *In vivo* studies

The effect of quercetin on tumor growth was analyzed *in-vivo* by angiogenesis and metastasis. Prostate tumor inhibition by quercetin *in vivo* in a mouse model is upregulated by the expression of Thrombospondin-1, which inhibits tumor propagation (Yang F. et al., 2016). (Zhao et al., 2016) reported that quercetin inhibited angiogenesis by mediating the calcineurin/NFAT pathway in the BALB/c mice breast cancer model. Quercetin’s inhibitory effect on metastasis is depicted by *in-vivo* experiments. Quercetin suppresses EMT by interfering with EGFR’s signaling pathway, thereby reducing VEGF expression (Jia et al., 2018).

### 3.2 Anticancer mechanisms of epigallocatechin gallate

The antitumor activity of EGCG is attributed to its capacity to mediate signaling pathways and regulate cells’ undesired survival and proliferation. The cell death by EGCG is initiated by the intrinsic pathways in various cancers (Table 1).

EGCG suppresses ERK1/2, NF-κB, and Akt-mediated signaling and activates p53 and PTEN/p21 for cell apoptosis along with the alteration of the Bcl-2 protein ratio (Almatroodi et al., 2020). Induction of apoptosis takes place through some pathways which incorporate intrinsic and extrinsic pathways, regulatory proteins, the strain on the endoplasmic reticulum through the activation of caspase-mediated pathways, death receptors, downregulation of several anti-apoptotic proteins, upregulation of Bad, and Bax pro-apoptotic proteins in human adrenal cancers cells (Wu et al., 2019) (Figure 2). The cell cycle arrest is triggered by EGCG at G0/G1 phase and occurs via the regulation of several cyclins in pancreatic cancer cells.

In cyclins, EGCG stops cyclin D1 and turns on p21, which in addition consists of ERK, IKK, and PI3K signaling pathways in which colorectal cancer cells are being inhibited from proliferating (Rady et al., 2018) (Figure 2). In cervical cancer, EGCG prevented the spread, invasion, and migration of HeLa cells via down-and upregulation of MMP-9 and TIMP-1 genes, respectively (Sharma et al., 2012). EGCG inhibited tumor growth by activating VEGF/VEGFR axis, interrupting the HIF-1a expression and other foremost growth factors, inactivation of PAR2-AP, ERK1/2, and NF-κB pathways were blocked (Zhou et al., 2012). In esophageal tumor cells, EGCG suppresses cellular viability through the reduction of p-ERK1/2, c-Jun, and COX-2, which in addition reasons the activation of caspase-3 in conjunction with the suppression of the COX-2 expression (Ye et al., 2012). EGCG significantly suppresses the glycolytic enzymes’ mRNA levels in breast cancer cells (Wei et al., 2018). In colorectal-cancer-cell, EGCG activates caspase-3 for apoptosis, PARP, downregulation of STAT3 and phosphorylated STAT3 (p-STAT3), decreased Bcl-2 MCL-1, vimentin, along with the increase in E-cadherin (Luo et al., 2021).

EGCG set up its important function for inhibition of glioma and showed Mitogen-activated protein kinase pathway (MAPK) involvement in apoptosis and proliferation (Figure 2). EGCG treatment with TRAIL induces rapid apoptosis which could be a striking approach for treating several gliomas (Li et al., 2014).

#### 3.2.1 *In vitro* studies

EGCG possesses an anti-tumor effect on various cancer cells *in-vitro,* including adrenal and breast. Cervical, colorectal, gastric, liver, lung, ovarian, prostate, and skin cancer cells (Gan et al., 2018). (Huang et al., 2017) stated that EGCG suppressed breast cancer cell proliferation in a concentration-structured manner. The comparison of expression of P53 in the EGCG-combined with the si-P53 group revealed the enhanced expression of the former in the combination of EGCG than that of the si-P53 group. (Luo et al., 2014) reported a dramatic decrease in cell growth in breast cancer cells on treatment with a range of quantities of EGCG in comparison with control cells. Moreover, expression of HIF-1α protein in addition to vascular endothelial growth factor declined in cancer cells in a dose-dependent approach on pre-treatment with progressing concentrations of EGCG. In ovarian cancer cell growth, EGCG was reported to show inhibition of growth in all cell lines in a dose-dependent manner; thereby, apoptosis and cell cycle arrest was triggered (Kim et al., 2004). (Wu et al., 2019) reported that EGCG inhibited thyroid carcinoma cells’ viability and proliferation as well as cell cycle progression.

Another study suggested that the pro-EGCG remedy affected tumor angiogenesis in endometrial cancers. Pro-EGCG contributes to the tumor angiogenesis inhibition in xenograft animal models which takes place through downregulation of vascular endothelial factor A, and HIF-1 α. Pro-EGCG remedy reduced vascular endothelial factor A secretion from endometrial cancer cells. In EGCG-treated endometrial adenocarcinoma, the expressions of the sex hormones estrogen and progesterone receptor were decreased along with the decrease in MAPK signals and phospho-Akt. Further over, EGCG arrested cells in the G0/G1 cell cycle phase (Park et al., 2012). An interesting study to assess the characteristic of EGCG effects on the metabolism of pancreatic adenocarcinoma cells was undertaken. Results found that the adenocarcinoma cells treated with catechin enormously suppressed lactate production and anaerobic glycolysis in addition to glucose consumption. A pioneering examination confirmed the blended better antitumor reaction of curcumin plus EGCG on prostate cancer cells, which in any other case had been immune to chemotherapy, and apoptosis inducers. The co-remedy of EGCG and curcumin enhanced the protein expressions of p21. However, their expression was unchanged on treatment with each compound alone (Eom et al., 2015).

An *in-vitro* look on bladder cancer cells showed that EGCG prompted morphological modifications and inhibition in a dose- and time-established fashion. In addition, EGCG-treated with bladder tumor cells found sub-G1 populations similarly to activation of caspase-3 and -9. Bladder cancer cells’ proliferation was decreased and controlled by EGCG treatment *in vitro* migration, followed by the downregulation of N-cadherin and Akt signaling inactivation (Jankun et al., 2014).

#### 3.2.2 *In vivo* studies

*In-vivo* findings tested that EGCG brought about cell arrest in the G1 phase, and apoptosis was induced via ROS generation (Lu et al., 2015). The intraperitoneal injection of EGCG inhibited the gastric cancer cells’ growth and declined the expression of mRNA of endothelial growth factors in tumor cells in a dose-dependent fashion (Cheng et al., 2020). (Sur et al., 2016) evaluated the chemopreventive and therapeutic efficacy of EGCG in combination with theaflavin on hedgehog pathways during CCl4/N-nitosodi-ethylamine-induced mouse liver carcinogenesis. Findings revealed that both the compounds in combination limited the hepatocellular carcinoma development at the 30th week after the carcinogen had been given, depicting their potential chemopreventive action in the continuous treated group than in the post-treated group. EGCG/theaflavin remedies precipitated reduction in apoptosis and proliferation, respectively. EGCG has a defensive effect on the growth, liver, and pulmonary metastases of colon cancers in nude mice by the activation of the Nrf2-UGT1A signal pathway (Almatroodi et al., 2020).

A phase I study on the safety and effectiveness of EGCG mouthwash was performed, which was given in addition to radiation in head and neck cancer episodes. The assigned dose of EGCG mouthwash was directed in a standard 3 plus 3 dose-escalation design. EGCG administration decreased oral mucosal injury in patients (Zhu et al., 2020).

### 3.3 Anticancer mechanisms of curcumin

Curcumin activates the formation of ROS, enhances intracellular calcium levels, and changes cell membrane potential, which further activates apoptotic pathways in tumor cells (Liu and Ho, 2018). Curcumin disrupts the maintained stability of the mitochondrial membrane, inflicting the elevated suppression of the Bcl-2 and Bcl-xL apoptotic protein expression (Tomeh et al., 2019). Curcumin’s anticancer mechanism involves a decrease in glucose absorption and lactate generation (Warburg effect) in cancer cells which takes place through downregulation of pyruvate kinase-M2 (PKM2), executed via suppression of the rapamycin-hypoxia-inducible issue 1α (Tomeh et al., 2019). Overall, the anti-tumor activity of curcumin in the cell causes an increase in growth suppressor factors, decreases associated proliferative pathways, and Wnt-βcatenin, acts on angiogenesis (VEGF), and enhances apoptosis (Allegra et al., 2017) (Table 1).

#### 3.3.1 *In vitro* studies

Curcumin has anti-cancer properties against breast cancer on Her2-positive cell lines (SKBR3 and BT474), having a lower inhibitory concentration (IC_50_) for curcumin compared to triple-negative cell lines. The decrease in IC_50_ is directly proportional to the expression of ER rather than Her2. ER-negative cell lines, i.e., SKBR3 and MBA-MB-231, have a lower IC_50_ for curcumin than ER-positive cell lines like BT474 and MCF7 (Liu and Ho, 2018). When treated with curcumin, the malignant colorectal cells reduce M(1)G levels without any significant change in COX-2 protein levels. Treatment of HCT 116 colorectal cancer cells with curcumin pronounced the arrest of the cell cycle in the G2/M phase via the miR-21 gene together with the inhibition of the proliferation of tumor cells.

Treatment of curcumin on human mammary epithelial and MCF-7 breast cancer cells reported a substantial decline in telomerase activity in a concentration-dependent manner associated with the downregulation of hTERT (Tomeh et al., 2019). The growth of oral mucosal epithelial cell lines and squamous cell carcinoma was inhibited by curcumin with the least consequence on normal oral epithelial cells. Curcumin reduced the efficiency of the eIF4F translational complex of mucosal cells via suppressing phosphorylation of eIF4G and eIF4B, combined with a decrease in total levels of eIF4E and Mnk1. Antitumour effects of curcumin were found to be effective in another investigation in oral cancer cells, which triggered the promoter activity of insulin-like growth factor binding protein-5 (IGFBP-5) and CCAAT/enhancer-binding protein alpha (C/EBP-α).

Curcumin triggers p53-established cell demise in basal cell carcinoma and breast cancer. However, in neuroblastoma, mammary epithelial carcinoma, and colon cancer, curcumin remedy unexpectedly via upregulated p53 expression, triggered nuclear translocation of p53, accompanied by p21 and Bax expression induction (Allegra et al., 2017; Giordano and Tommonaro, 2019). Curcumin in tumor tissue enhances the p53 molecule expression, modulates the pathway responsible for apoptosis in ovarian cancer as well as induces the p53 expression in nasopharyngeal carcinoma by mediating serine phosphorylation of p53 (Sultana et al., 2021).

#### 3.3.2 *In vivo* studies

The inhibition of IGFBP-5 and C/EBP-alpha by curcumin was mediated through p38 activation and caused a decline of tumorigenesis in a mouse xenograft model *in vivo* (Wilken et al., 2011). (Kocaadam and Şanlier, 2017) examined the antitumor property of curcumin in various head and neck squamous carcinoma cell lines, CCL23 (laryngeal), CAL27, UM-SCC14A, and UMSCC1 (oral). Curcumin decreased the expression of NF-κB and controlled the expression of gene products, phospho-IκB-α, as well as inhibition of nuclear localization (Kocaadam and Şanlier, 2017). *In-vivo* studies on mice tormented by colorectal cancer showed a better reaction to radiation remedy in aggregation with curcumin owing to its assets to target nuclear factor (NF-κB) (Tomeh et al., 2019).

### 3.4 Anticancer mechanisms of silibinin

Silibinin inhibits the cell’s metabolic activity and induces apoptosis via caspase 3 and PARP-1 regulation in a concentration- and time-dependent fashion. Silibinin induces autophagy upregulation of protein which was associated with microtubule formation (Bai et al., 2018). Silibinin reduced tumor growth by downregulating extracellular signal-regulated kinase and Akt in human ovarian cells (Cho et al., 2013). Silibinin triggered autophagic cell death in breast cancer cells; the usage of mitochondrial potential became a result of ROS formation and ATP depletion, causing stimulation of expression of Bcl-2 adenovirus E1B 19-kDa-interacting protein 3 (Jiang et al., 2015). Together, apoptosis and autophagy were strongly induced by silibinin through the interference of various pathways, up- and downregulation of the expression of caspase-3, Atg5, Atg7, Bcl-2, COX-2, HIF-1α, VEGF, MMP-2, 9, respectively (Sameri et al., 2021) (Figure 2) (Table 1).

#### 3.4.1 *In vitro* studies

In human hepatocellular carcinoma (HCC), silibinin powerfully repressed the growth of HepG2 and Hep3B cell lines and showed relatively more potent cytotoxicity in Hep3B cells, causing apoptosis induction. In HepG2, silibinin caused G1and G2-M phase arrest in Hep3B cells.

A study conducted by (Mateen et al., 2010) demonstrated the effect of silibinin on non-small cell lung cancer (NSCLC) subtypes and bronchoalveolar carcinoma cells. The cell growth was affected with silibinin treatment, and the cell cycle was reported to arrest at the G1 phase in a dose and time-dependent manner. Silibinin decreased kinase expression in all of the cell lines; however, no impact was found on CDK4 activity in H460 cells, with the concomitant reduction in retinoblastoma protein phosphorylation. Silibinin strongly caused a decline in cell viability and death in MCF-7 cells related to expanded p53 expression (Noh et al., 2011). (Kaur et al., 2010) studied the effect of treatment of silibinin on the expansion of tumorous human CRC cells in culture as well as in xenograft model. Results concluded that silibinin treatment in SW480 inhibited cell growth, prompted cell death, and reduced nuclear-cytoplasmic β-catenin; however, no longer in wild type, depicting its effect on the β-catenin pathway along with related biological responses. Anticancer properties of silibinin investigated on CT26 mouse colon cell lines showed a reduction in proliferation, cell survival, angiogenesis, and migration. Silibinin significantly reduced CT26 cells’ survival along with up-and downregulation of various signal pathways (Sameri et al., 2021).

*In-vitro* studies have demonstrated that silibinin mediates Kip1/p27 declines cyclin D1, D3, cyclin-dependent kinase (CDK)-2, and CDK-4 expressions in both the cell lines. In Hep3B cells, silibinin reduces the protein concentrations of G2-M regulators with the inhibition of CDK-2, CDK-4, and CDC2 activity in HCC cells (Varghese et al., 2005).

#### 3.4.2 *In-vivo* studies

(Cho et al., 2013) reported that the silibinin *in vitro* and *in vivo* reduces tumor growth via downregulation of the extracellular signal-regulated kinase (ERK) and Akt in human ovarian cancer cells. Silibinin, when given orally to A2780 cells, shrinks the tumor volume, decreases Ki-67-positive cells, increases transferase-mediated dUTP nick end labelling -positive cells, caspase-3 activation, p-ERK, and p-Akt inhibition.

### 3.5 Anticancer mechanisms of apigenin

Apigenin triggers cell cycle arrest at various proliferation laps, regulates intrinsic apoptotic pathways, and promotes different anti-inflammatory pathways (Iizumi et al., 2013; Lapchak and Boitano, 2014). Apigenin enhances the expression of anti-oxidant enzymes plus induces the inhibition of metastasis and angiogenesis (Rezai-Zadeh et al., 2008) (Table 1).

Various signaling pathways are modulated by apigenin in tumor cells which include phosphoinositide 3-kinase and Wnt/β-catenin pathways (Yan et al., 2017). Apigenin upregulates the Bad and Bax, which are pro-apoptotic proteins, and/or downregulates pro-survival associates, i.e., Bcl-xL and Bcl-2, thus activating the apoptotic process within the cell. Cancer cell proliferation is inhibited using apigenin through modulation of the cell cycle and blockage at checkpoints, i.e., G2/M or G0/G1, that is related to the suppression of cyclin B1 and its activating partners, together with the increase of expression of cell cycle inhibitors, ROS accumulation and hence, DNA damage (Lee et al., 2014) (Table 1). In cervical cancer, apigenin changes Bax/Bcl-2 ratio and triggers apoptosis, followed by inhibition of the Raf/MEK/ERK signaling pathway (Zhang et al., 2019). Autophagy is induced through AMPK activation, which is triggered by apigenin, and the mTOR signaling pathway is also inhibited (Sung et al., 2016).

#### 3.5.1 *In vitro* studies

(Zhang et al., 2019) examined the anticancer property of apigenin on human cervical cancer cells. Apigenin at IC_50_ of 15 µM suppressed the colony formation of HeLa cells, altered the Bax/Bcl-2 ratio, triggered apoptosis, and altered the Raf/MEK/ERK signaling pathway. (Zhang et al., 2008) reported that apigenin triggered blockage of the cell cycle in addition to apoptosis and inhibited the expression of cyclin B1 in KYSE-510 and OE33 cells. In KYSE-510 cells, apigenin showed induction of expression of p21/waf1 and promoted G2/M phase cell cycle arrest in KYSE-510 and OE33cells. Higher PIG3 mRNA and protein expression due to apigenin processing indicated the involvement of PIG3 in apigenin-induced apoptosis in both KYSE-510 and OE33 cells. The increase in activity of caspase-3 and -9 fragments recommended that PIG3 induction was mediated through apigenin which further caused esophageal cancer cell apoptosis via interfering with the mitochondrial pathway.

(Masuelli et al., 2011) revealed that apigenin triggered tumor cell death in tongue, pharyngeal cancers, and cancer in FaDu cells by destabilization of the EGFR/ErbB2 signaling pathway. In head and neck squamous carcinoma cells, apigenin downregulates the phosphorylation of EGFR and ErbB2 at precise sites and is regarded to be associated with the stimulation of Ras/Raf/Erk-1/-2 and Akt pathways. Apigenin treatment causes arrest of cell growth and induces apoptosis in many tumors by the modulation of diverse signaling pathways. The authors also analyzed the interactions between apigenin and TRAIL in NSCLC cells. Results demonstrated apigenin and TRAIL produced a synergistic effect for the induction of apoptosis of NSCLC cells by upregulating death receptors-4, and -5 in a p53-dose-dependent fashion. Additionally, the pro-apoptotic proteins were upregulated, while the anti-apoptotic proteins, viz. Bcl-xl and Bcl-2 were reported to be downregulated. Apigenin inhibited the activation of NF-κB, AKT, and ERK.

#### 3.5.2 *In vivo* studies

In the mouse xenograft model, the combined treatment of apigenin and TRAIL entirely inhibited tumor proliferation as compared to the treatment of apigenin or TRAIL alone. In total, apigenin enhanced antitumor activity in NSCLC cells which was induced by TRAIL through the inhibition of several pro-survival regulators.

### 3.6 Anticancer mechanisms of luteolin

Luteolin inhibits the progression of cancerous cells, protects from carcinogenic stimuli, activates cell division blocks, and helps in the initiation of cell death via activating numerous signaling pathways. Luteolin also reverses the epithelial-mesenchymal transition (EMT) by altering a mechanism that involves cytoskeleton contraction resulting in a change in the expression of epithelial biomarker E-cadherin in addition to a decrease in expression of the mesenchymal biomarkers. In addition, luteolin elevates the intracellular concentration of ROS via the stimulating ER stress, which is lethal as well as mitochondrial dysfunction in glioblastoma cells (Imran et al., 2019). Modulation of ROS levels, topoisomerase I and II, P13K, NF-kappaB and AP-I activity reduction, and p53 stabilization are mechanisms contributing to the anti-tumor activity of luteolin (Lopez-Lazaro, 2009). Luteolin inhibits specific critical cancer, which is followed by the activation of various signaling pathways like mTOR and MAPK. Programmed cell death is mediated through the cell cycle arrest. In colon cancer, cells treated with luteolin block the cycle of the cell at the G2/M phase. Proteins involved in G2/M transition, specifically cyclin B and CDC2, were downregulated, whilst upregulated proteins were CDK2 and cyclin A. Luteolin arrests the G2 phase in non-small-cell lung cancer cells by the inhibition of the cyclin A expression plus CDC2 phosphorylation (Ganai et al., 2021).

#### 3.6.1 *In vitro* studies

*In vitro* studies on luteolin have revealed that luteolin inhibits breast cancer cell proliferation, is stimulated by IGF-1, arrests cell cycle development, plus induces cell death in a time-and dose-dependent manner (Table 1). Luteolin declined IGF-1-stressful Erk1/2 phosphorylation depicting its inhibitory effect. In breast cancer cells, luteolin increases or decreases the expression of numerous estrogen stimulating genes and cell cycle pathway genes and modifications histone H4 acetylation that’s positioned on the PLK-1 promoter (Yang et al., 2014). In colon cancer, luteolin exhibits a withdrawing effect on nitric oxide synthase (iNOS) and COX-2 expressions and a suppressing one on MMP-2 and -9 expressions. Recent research has discovered that treatment with dimethylhydrazine induced renal bleeding in addition to colon polyps in rats with the enhancement in COX-2 and oxidative stress. Luteolin treatment declined the concentrations of iNOS and COX-2 (Abdel hadi et al., 2015).

Luteolin treatment (15 μM, 24 h) to human pancreatic cancer cells significantly declined nuclear GSK-3β and NF-κB p65 expression (Bothe et al., 2010). The chemopreventive plus chemotherapeutic actions of luteolin against prostate cancer were demonstrated in the vastly persistent Du145-III prostate cancer cells. The study reported that luteolin decreased the growth of these cells and suppressed cancer cell intrusion. (Johnson et al., 2015) reported that luteolin hampered PCa cell growth that was androgen-sensitive and independent, caused the downregulation of miR 301, and triggered cell death in LNCaP PC3 cells.

In prostate fibroblastoma, luteolin caused inhibition of myofibroblast phenotypes and extracellular matrix contraction, which was induced by TGF-β; suppression of TGF-β signaling involving activation of AKT and ERK; suppressed activation of RhoA (Johnson and Mejia, 2013) (Table 1).

A recent study by Chakrabarti and Ray (2016) investigated the consequence of taking two different luteolin concentrations, viz. 25 and 50 μM, on the neuroblastoma cells in a dose-dependent manner. Luteolin aroused apoptosis, triggered G0/G1 cell cycle arrest, plus lowered the mitochondrial membrane potential. Luteolin in oral cancers affected the phosphorylation of ataxia-telangiectasia in conjunction with pathways of DNA repair. Luteolin reduced the growth of the SCC-4 cells and multiplied the death of tumor cells by interfering with the expression of some cyclins, cyclin-established kinase (CDKs) (Wang F. et al., 2015). In head and neck squamous cell carcinoma, luteolin inhibited the tumor expansion plus histone acetylation, promoted arrest of the cell cycle, declined the movement of cells as well as altered gene expression, and upregulation of p53 induced plus miRNA systems (Tu et al., 2016).

Luteolin played an influential part in human NSCLC cell line A549 against tumor cell propagation by inducing cell death along with suppression of movement of cells. Apoptosis induction involves numerous steps in cells, viz. Caspases activation, alteration of MEK phosphorylation, Bcl-2 family proteins expression and downstream of kinase ERK, along with Akt phosphorylation (Figure 2). The luteolin’s anticancer properties were well explained on NCIH460 cells via induction of cell-death that was mediated by Sirt1 (Imran et al., 2019).

*In-vitro* studies on esophageal carcinoma cells demonstrated that antitumor activity of luteolin on cancer cells occurred through the activation or suppression of several mechanisms which included the activation of apoptotic cell death plus caspase-3, and induction of arrest of the cell cycle at the G2/M phase (Ganai et al., 2020; Ganai et al., 2021). Luteolin also lowered the mitochondrial membrane potential and enhanced the regulatory protein of the cell cycle in addition to the increase in the concentrations of apoptosis-related proteins.

#### 3.6.2 *In vivo* studies

In a recent study, azoxymethane (AOM) intraperitoneal administration triggered colon carcinogenesis in Balb/C mice via an increase in the levels of tumor markers. Oral administration of luteolin appreciably declined the concentrations of tumor markers as well as expressions of MMP-2 and -9 (Naso et al., 2016). In pancreatic most cancers cells, *in vivo* apoptosis was brought about by luteolin through the suppression of the K-ras/GSK-3β/NF-κB pathway with the release of cytochrome C, caspase-3activation, and decline in Bcl-2/Bax ratio (Pandurangan et al., 2014). In a xenograft mouse model, at a specific dose, luteolin reduced the pro-inflammatory cytokines and TNF-α stages in PC3 cells similarly to decreased weight and extent of tumors, affected the cell capability, triggered cell death, and downregulated the ERK, AKT, mTOR, MMPs expressions (Han et al., 2016) (Figure 2).

In another study, in a xenograft model in mice with NSCLC cells, luteolin and Infra-Red radiation co-treatment activated apoptotic cell death, and declined the expression of B-cell lymphoma 2 (Bcl-2) along with caspase-complex activation. Also, luteolin inhibited the progression of xenograft mouse models of esophageal squamous cell carcinoma (Wu et al., 2015).

### 3.7 Anticancer mechanisms of genistein

Genistein possesses antitumor activities by the modulation of multiple signaling pathways, i.e., extracellular signal-regulated kinase 1/2 (ERK1/2), protein-tyrosine kinase (PTK), nuclear transcription factor-kB (NF-κB), mitogen-activated protein kinase (MAPK), and phosphoinositide 3 kinase/Akt (PI3K/Akt), matrix metalloproteinases (MMPs) and, Bcl-2-related X protein (Bax) (Lee et al., 2012; Spagnuolo et al., 2015; Bhat et al., 2021) (Figure 2). Genistein at higher concentration results in apoptosis mediated by inhibition of various proteins associated with primary tumor growth, i.e., cell cycle regulators (cyclin class) and the Akt family of proteins. However, when genistein is given at a lower concentration via diet inhibits the transforming growth factor (TGF) pathway, which affects pro-metastatic actions such as cancer cell detachment and invasion (Pavese et al., 2010). Genistein induces endoplasmic reticulum (ER) stress by upregulation of glucose-regulated protein 78 (GRP78) and CHOP expression. ER stress inhibitor also enhances genistein-induced cell death (Yang F. et al., 2016) (Table 1).

#### 3.7.1 *In vitro* studies

*In vitro* studies of genistein revealed that in ovarian cancer cells, it triggers cell cycle arrest in the G2/M phase, and induces caspase-3 activity hence, enhances the apoptotic population. As the genistein concentration increases concurrently after 48 h of exposure, an increase in cell proportion in the G2/M phase was also noticed (Choi et al., 2007). Genistein in HO-8910 cells changed the protein levels, which are related to the checkpoint pathway, resulting in the inhibition of cancer cell propagation (Ouyang et al., 2009). Pretreatment with genistein by Solomon et al. (2008) in platinum-sensitive and -resistant ovarian cancer cells inhibited cell growth by NF-κB inactivation.

A recent study (Yang F. et al., 2016) reported that in cervical cancer cells, cell death triggered by genistein is mediated by endoplasmic reticulum (ER) stress and suppresses the HeLa cells’ viability in a dose-dependent fashion. Additionally, genistein also played an important part in triggering ER stress in HeLa cells mediated through the upregulation of glucose-regulated protein 78 and CHOP expression. Genistein helps in the initiation of cell death in several hepatocellular carcinoma cells (HCCs). Genistein exhibits potential anti-invasive and anti-metastatic activities against 12-O-tetra decanoyl phorbol-13-acetate-mediated metastasis which proceeds through downregulation of MMP-9 and NF-kB, and activator protein 1 transcription factors occurring via inhibition of MAPK (Spagnuolo et al., 2015).

Genistein triggered apoptosis in the low-invasive MCF-7 and the high-attack in breast cancer cell lines (10–100 mM) in a dose-dependent approach (Spagnuolo et al., 2015). Genistein declined the cell viability as well as the colony-forming potential of HepG2 liver cancer cells in a dose-and concentration-dependent manner (IC_50_ = 25 μM). Genistein triggered the ROS generation, thus, favoring cell death. Apoptosis induction by genistein is linked with the upregulation of cytosolic cytochrome c, with the downregulation of Bcl-2 in HepG2 cells (Zhang et al., 2019). Genistein significantly inhibits the propagation of hepatocellular carcinoma and induces death of the cell in a concentration and time-dependent manner. During genistein treatment, the reported enhanced percentage of cell death at a concentration (20 μM) with the progression of time 24, 48, and 72 h were 35, 42, and 65%, respectively (Sanaei et al., 2018).

#### 3.7.2 *In vivo* studies

Genistein administration in ovariectomized rats increased uterine weight. An antagonistic effect of genistein on the estradiol increases uterine epithelial height (Diel et al., 2006). A protective effect of genistein against the progression of endometrial cancer along with atypical endometrial hyperplasia in mice was observed by the modulation of the expression of genes known to be linked with estrogen plus cytokines (Lee et al., 2012). Genistein significantly suppressed the attack of Bel 7,402 cells and altered the cell cycle, cell death, and angiogenesis in the renal parenchyma of nude mice with a xenograft transplant. In treating mice with genistein, tumor growth was significantly slowed down in male BALB/C nu/nu mice that were injected with Bel 7,402 cells (Gu et al., 2005).

### 3.8 Anticancer mechanisms of protocatechuic acid

Protocatechuic acid (PCA) exerts pro-apoptotic and anti-proliferative properties in different tissues. Anti-tumor property of PCA involves the stimulation of c-Jun N-terminal kinase, p38 subgroups of the mitogen, which are further activated by the protein kinase (MAPK) family (Khan et al., 2015). PCA blocks the retinone-induced apoptotic death of cells. PCA influences the activity of cyclooxygenase (COX) inducible isoenzyme as well as nitric oxide synthase along with regulating proteins of the cell cycle, or inflammatory cytokines, comprising the part of oncogenesis (Kakkar and Bais, 2014). Another mechanism of action for the anti-cancer activity of PCA follows the downregulation of the Ras/Akt/NF-kB pathway by targeting activation of RhoB, further leading to a drop in MMP-2-mediated cellular actions in cancer cells (Lin et al., 2011). In five cancer cells, concentration-dependent PCA lowered cell viability, increased lactate dehydrogenase leakage due to increased DNA fragmentation, lowered mitochondrial membrane potential, and declined the Na^+^-K^+^-ATPase activity; thus, elevated caspase-3 and caspase-8 activities, therefore, promoted cell death in cancer cells (Yin et al., 2009) (Table 1).

#### 3.8.1 *In vitro* studies

The apoptotic effects of PCA on different types of cancers, namely, lung cancer, human breast cancer, and prostate cancer cell, in a dose-dependent approach (1, 2, 4, 8 μmol/L) were studied. The authors concluded that PCA declined cell feasibility, increased leakage of lactate dehydrogenase, DNA fragmentation, suppressed mitochondrial membrane potential, and decreased Na^+^-K^+^-ATPase activity. Although PCA elevated caspase-3 activity in 5 cancer cells still, at the concentration of 2–8 μmol/L, PCA considerably enhanced caspase-8 activity, lowered intercellular adhesion molecule expression in cancer cells, and declined vascular endothelial growth factor production. Additionally, PCA reduced interleukins (IL)-6 and IL-8 in cancer cell lines (Yin et al., 2009).

#### 3.8.2 *In vivo* studies

Metastasis mouse models *in vivo* were analyzed to study the effect of PCA on cancer cell attacks. The study concluded that PCA at non-cytotoxic concentrations inhibited cell migration and invasion. PCA treatment caused decreased expression of MMP-2 subsequently, a rise in MMP tissue inhibitor. PCA-inhibition of MMP-2 activity expression was consorted by NF-kB inactivation, which was further mediated via RhoB/protein kinase C and Ras/Akt cascade pathways. Taken together, PCA reduced the progression of B16/F10 melanoma cells in mice (Lin et al., 2011).

### 3.9 Anticancer mechanisms of rosmarinic acid

RA’s antitumor mechanism of action involves significant suppression of cell viability, progression, invasiveness, and migration thus, induction of cell death. Anti-tumor effect of RA occurs via regulation of the miR-506/MMP2/16 axis in pancreatic tumor cells. The treatment of RA suppresses the tumor expansion of pancreatic cells and enhances and suppresses the expression of miR-506 and MMP2/16/Ki-67, respectively (Han Z. et al., 2018). RA possesses an inhibitory effect on melanoma cells’ proliferation, movement, and invasion via inhibiting expression of the ADAM17/EGFR/AKT/GSK3β axis (Huang et al., 2021). In colorectal cancer, the anticancer effect of RA causes the inhibition of COX-2 activation by repression of binding of activator protein-1 (AP-1) and c-Fos inducing agents (Hossan et al., 2014). The anti-metastatic effect of RA occurs via the AMPK phosphorylation, and colorectal cancer proliferation is slowed down, which further proceeds by the initiation of cell cycle arrest and apoptosis, followed by inhibition of expression of MMP-2 and MMP-9. In conclusion, RA effects on EMT and MMPs expressions result in AMPK activation (Han Z. et al., 2018). In leukemia, RA suppresses the activation of NF*-k*B-dependent anti-apoptotic proteins via phosphorylation inhibition, I kappa-B-alpha degradation, and p50 and p65 nuclear translocation (Moon et al., 2010) (Figure 2).

#### 3.9.1 *In vitro* studies

The effects of RA on pancreatic tumor progression and its primary molecular mechanisms were explored by Han et al. (2019) (Table 1). Authors concluded that RA considerably suppressed vital cellular functions in pancreatic tumor cells, finally inducing cell death in pancreatic cells. Further, RA significantly up-regulated and knocked down the expression of miR-506 in pancreatic cancer cells, accompanied by the suppressive effects of RA on cell growth and EMT, and prohibited increased impact of RA on cell apoptosis in pancreatic tumor cells. Additionally, RA treatment concealed the MMP2/16 expression in pancreatic cancer cells.

In another study, anticancer effects of RA in A375 cells melanoma cells via downregulation of ADAM17 and expression of ADAM17/EGFR/AKT/GSK3β were also evaluated. Results revealed that A375 cells treated with RA had decreased cell viability, propagation, invasive abilities, melanin content, and decreased expression of MMP-2 and MMP-9 proteins as compared to normal cells. RA increased the expression of pro-apoptotic proteins and suppressed Bcl-2 expression (Huang et al., 2021). (Han Z. et al., 2018) reported that RA inhibited the metastatic properties of CRC cells through the phosphorylation of AMPK. RA suppressed the CRC cells propagation, brought about cell cycle arrest, and hence, initiated apoptosis. RA mediated EMT through the growing the expression of an epithelial marker, E-cadherin, and lowering the expression of the mesenchymal markers and N-cadherin (Figure 2). RA treatment caused CRC cells’ invasion and migration suppression and declined the expressions of MMP-2 and MMP-9.

In human leukemia U937 cells, RA was reported to trigger apoptosis which was in turn induced by TNF-α and generation of ROS. RA inhibited NF-kB activation via phosphorylation inhibition, followed by the degradation of IκBαand nuclear translocation of p50 and p65, which is associated with NF-kB-dependent anti-apoptotic proteins suppression (Hossan et al., 2014). RA inhibited the movement of human bone-homing breast cancer cells suggesting that RA affected the bone metastasis in breast carcinoma via activation of the NF-kB ligand (RANKL)/RANK/osteoprotegerin (OPG) pathway with the simultaneous repression of the IL-8 expression (Xu et al., 2010).

#### 3.9.2 *In vivo* studies

*In vivo* studies have exposed that RA inhibited the tumor growth dose-dependently of pancreatic cancer cells and enhanced the expression of miR-506, although lowered the MMP2/16 and Ki-67 expression in xenograft nude mice. Mice treated with oral RA suppressed tumor induction NF-κB, TNF-α, VEGF serum, and VEGF receptors. RA induced apoptosis by restoring the expressions of Bcl-2, Bax, and caspase-3. In Ehrlich solid tumor mice, RA, in combination with paclitaxel, significantly decreased the growth of the tumor with enhancement in levels of apoptotic markers (Mahmoud et al., 2021).

In the swiss albino xenograft model, RA administration suppressed tumor formation, positively altered the antioxidant level, and stabilized the 7,12-dimethyl-benz(a)anthracene (DMBA)-induced alterations in the apoptotic markers (Sharmila and Manoharan, 2012).

### 3.10 Anticancer mechanisms of chlorogenic acid

Mechanism of action of CA involves induction of apoptosis upregulation of p21 leading to G0/G1 cell cycle arrest, reducing the potential of the mitochondrial membrane of the cell resulting in mitochondrial dysfunction, and escalating the activation of the caspase-3 pathway, ultimately causing cell death (Dai et al., 2012; Huang et al., 2019). The apoptosis effect of CA occurs via the increase in p53 and Bax protein expression with a decrease in Bcl-2 expression followed by activation of the caspase-3 pathway (Changizi et al., 2020) (Figure 2). The BH3-protein Bcl-2 binding component 3 (BBC3) is upregulated by CA, which causes mitochondrial death (Jiang et al., 2021). CA inhibits the migration and invasive capacity of tumor cells, modulates mTOR2-related pathways, and reduces the phosphorylation of p-protein kinase C alpha (PKCα), Akt, along with declined expression of Rictor and F-actin (factors responsible for cell growth and organization of actin cytoskeleton). Directly involves the mTOR2/F-actin pathway for tumor suppression (Tan et al., 2019). CA inhibits the NF-B/EMT signaling pathway, concluding its anticancer and antimetastatic properties (Zeng et al., 2021).

#### 3.10.1 *In vitro* studies

In human hepatoma cells (HCC), CA dose-dependently inhibits the activity of cell lines, i.e., Hep-G2 and Huh-7. CA alters the NF-κB signaling pathway and turns on the mitochondrial cell death of HCC through the upregulation of (BBC3) (Jiang et al., 2021). The inhibitory effect of CA *in-vitro* (A549 and HepG2 cell lines) and *in vivo* (female BALB/c nude mice) on tumor cells and its associated possible mechanisms engaged in filamentous actin (F-actin) organization was also investigated. Results demonstrated that CA effectively inhibited the increase of tumor cells.

A study conducted by Changizi et al. (2020) analyzed the effect of CA on 4T1 breast cancer cells. Highest CA concentration up-and down-regulated Bax and Bcl-2, respectively, in 4T1 treated cells. Further, outcomes showed growth in the expression of P53 and caspase-3 for the duration of remedy in CA-handled 4T1 cells. CA inhibits proliferation and stimulates preprophase apoptosis, arrests the cell cycle at the G0/G1 segment in human acute promyelocytic leukemia HL-60 cells on the greatest dose of 10 µM (Liu et al., 2013).

The anti-cancer effect of CA was evaluated in another study by Huang et al. (2019). Treatment of tumour cells with CA caused elevation in SUMO1 expression via acting on 3′UTR and stabilization of mRNA. Elevated levels of SUMO1 resulted in c-Mycsumoylation, downregulation of the miR-17 family, and p21 upregulation causing the G0/G1 cell cycle seize.

#### 3.10.2 *In vivo* studies

*In vivo*, CA administration to mice bearing tumor cell-implanted xenografts inhibited the growth of the tumor. Alteration in various signal pathways includes modulation of mTORC2 associated signaling pathways, decreased phosphorylation of p-protein kinase C alpha (PKCα), Akt, declined expression of Rictor and F-actin known for the activation of cell growth and organization of the actin cytoskeleton indicating the involvement of mTORC2/F-actin pathway in tumor suppression induced by CA (Tan et al., 2019).

CA antitumor effect was evaluated in a recent study (Zeng et al., 2021) in a subcutaneous tumor mouse model possessing 4T1 cells. Authors discovered that CA noticeably slowed down the tumor growth with the prolongation of the survival rate of tumor-bearing mice. CA exerted antitumor and anti-metastatic consequences using a modification of the NF-κB/EMT signaling pathway.

### 3.11 Anticancer mechanisms of action of eupatorin

Eupatorin showed an antitumor effect on human breast cancer cells and mediated CYP1- metabolism. The cytotoxic effect of eupatorin was investigated on the human breast carcinoma cell line as well as another cell line that was extracted from normal mammary tissue, MCF-10A. Eupatorin blocks the cell cycle in the G2/M phase in CYP1-expressing cell line MDA-MB-468; however, the authors did not report any effect on MCF-10A cells; hence, no expression of CYP1 enzymes was observed. Together, the presence of CYP1 family metabolism resulted in the flavone eupatorin selective activation in breast cancer cells but not in normal breast cells (Androutsopoulos et al., 2008) (Table 1).

Razak et al. (2019) operating on human breast carcinoma strains concluded that eupatorin triggered apoptosis, suppressed invasion, migration, and angiogenesis of cell strains, i.e., MDA-MB-231 and MCF-7 cells through inhibition of the Phospho-Akt pathway, and cell cycle obstruction. Cytotoxic consequences of eupatorin have been located on both cells; however, they remained non-poisonous to the ordinary cells of MCF-10a in a time- and dose-structured approach. However, eupatorin was reported to be mild cytotoxic on both cells after 24 h of treatment. A lower dose of eupatorin (5 μg/ml) prompted apoptosis via the intrinsic pathway and caspase-9 in assessment to caspase-8. The eupatorin exerted arrest of the cell cycle in MCF-7 and MDA-MB-231 cells at Gθ/G1 in a time-hooked-up fashion. *In vivo* studies have revealed that eupatorin up-regulated pro-apoptotic genes; however, SMAC/Diablo inhibited the Phospho-Akt pathway. In another study (Pavlopoulou et al., 2015), reported that eupatorin suppressed cell proliferation in human tumor cells. Eupatorin blocks cells at the G2-M phase and triggers cell death, activating a couple of caspases cytochrome-*c* release and breakdown of poly (ADP-ribose) polymerase in human leukaemia cells. Eupatorin initiates the mitogen-activated protein kinases phosphorylation and cell apoptosis through c-jun N-terminal kinases/stress-activated kinases inhibition. Cell death via eupatorin is mediated via every apoptotic pathway and ROS mechanism.

#### 3.11.1 *In vitro* studies

In HeLa cells (HCT116 cells) human cervical carcinoma cells, cell cycle arrest started after 12 h of eupatorin treatment at the G2/M segment, followed by induction of apoptosis. Eupatorin down- and up-regulated cyclin D1 and B1 after 3 and 12 h of eupatorin treatment, respectively. Eupatorin lowered p53, p21, and Bax protein concentrations via initiation of the breakdown of caspase-3, -7, and poly (ADP-ribose) polymerase. Eupatorin-triggered p21 and Bax expressions which were p53 and Egr-1-pathways dependent, respectively. The authors concluded that eupatorin induced cytotoxicity in HeLa human cervical carcinoma cells, which was dysregulated via G2/M cell cycle blockage and triggered apoptosis via the activation of the p53-pathway (Lee K. et al., 2016) (Table 1).

## 4 Therapeutic perspectives on polyphenols as potential anticancer agents

Most polyphenols are present in foods in the form of esters, glycosides and polymers that cannot be absorbed in their native form in the human gastrointestinal tract (Sharifi-Rad et al., 2022; Semwal et al., 2022). Thus, before absorption of the molecules must be hydrolyzed by saliva, intestinal enzymes or need to be metabolized by the colonic microflora. Subsequently, to be absorbed, polyphenols are subjected to conjugation in intestinal cells and then methylation, sulfation and/or hepatic glucuronidation (Sharifi-Rad et al., 2021b). Thus, the structural integrity and proper functioning of the digestive tract are essential for the optimal absorption of polyphenols. The variability of the biological activity of polyphenols beyond the particularities of the human body also depends on the biological properties of the classes and subclasses of polyphenols (Rudrapal et al., 2022). For the anticancer effect, polyphenols can also be consumed in the form of natural supplements. In the case of approved natural supplements, they must be administered according to the recommendations made by the manufacturer, which can be found on the product packaging (Tsoukalas et al., 2019a; Tsoukalas et al., 2021b) Although obtained from 100% natural ingredients, these supplements can have unwanted effects if not administered correctly (Tsoukalas et al., 2019b; Tsoukalas et al., 2021a). Therefore, future translational studies are needed to determine the effective therapeutic dose in humans and the route of administration.

A therapeutic limitation is represented by the possibility of polyphenols interfering with the absorption of nutrients or drugs. For example, they can reduce the body’s ability to absorb iron, thiamine or folic acid (Cory et al., 2018). More research needs to be done to better understand these potential interactions. To increase therapeutic efficacy, nanotechnologies have been tried to increase the absorption and reduced bioavailability of some polyphenols, and numerous studies have confirmed this benefit. Food supplements with polyphenols do not endanger health, but there are uncertainties about the side effects of polyphenol supplements (Rana et al., 2022). Therefore, new studies are needed in the future to overcome the biopharmaceutical problems of polyphenols in cancer. Also, future research are needed to evaluate the isolated and particular effects of certain classes/subclasses of polyphenolic compounds on various types of cancer, respectively extrapolating the possible anticancer benefits to the general population or particular high-risk groups.

## 5 Conclusion

In the attention to experimental evidence stated and mentioned withinside the review, it is miles glaring that polyphenols display more capability than other compounds by blocking more than one target in pathways and mechanisms associated with cancer progression. Various *in-vitro* studies suggest that the polyphenols modulate Nrf 2 and NF-kB activation in cells and also influence the MAPK and PI3K function in the cells, thereby establishing a prominent role in cancer cell progression. In connection, the possibility of combining conventional drugs with polyphenols (in individual or combination) offers valuable advantages for developing more anti-cancer therapies with greater efficacy. Although, there is no doubt about the role of oxidation in the occurrence of mutations in DNA and the increased risk of cancer, the isolated action of a particular polyphenol or polyphenolic compounds, in general, is unlikely to have a distant effect on the gastrointestinal tract - for example, in the mammary gland or lungs. While data on the beneficial effects of polyphenolic compounds as anticancer agents, it has been observed that all these benefits depend not only on the food content of polyphenols, but especially on their bioavailability in the gastrointestinal tract, and their ability to be absorbed into the circulatory system to reach the cells where they manifest their protective effect. Nevertheless, the usage of dietary polyphenols as whole foods can be a promising aspect of cancer prevention.

## Abbreviations

Bax, Bcl2-Associated X Protein; Bcl-2, B-cell lymphoma 2; BCRP, Breast Cancer Resistance Protein; Cyt-c, cytochrome-c; CDH1, cadherin-1; IGFR, insulin-like growth factor receptor; MAPK, mitogen-activated protein kinase; MRP1, multidrug resistance protein1; mTOR, mammalian target of rapamycin; P13K/Akt, phosphatidylinositol 3-kinase/protein kinase B; p53, tumor protein p53; RTKs, receptor tyrosine kinases; RAS: rat sarcoma virus; TNF-α, tumour necrosis factor-alpha; VEGFR2, vascular endothelial growth factor receptor 2,.
